# Let’s Chat: On-Screen Social Responsiveness Is Not Sufficient to Support Toddlers’ Word Learning From Video

**DOI:** 10.3389/fpsyg.2018.02195

**Published:** 2018-11-13

**Authors:** Georgene L. Troseth, Gabrielle A. Strouse, Brian N. Verdine, Megan M. Saylor

**Affiliations:** ^1^Department of Psychology and Human Development, Vanderbilt University, Nashville, TN, United States; ^2^Division of Counseling and Psychology in Education, University of South Dakota, Vermillion, SD, United States; ^3^School of Education, University of Delaware, Newark, DE, United States

**Keywords:** contingency, relevance, social cues, symbols, video, video chat, word learning

## Abstract

Joint engagement with a speaker is one cue children may use to establish that an interaction is relevant to them and worthy of attention. People on pre-recorded video cannot engage contingently with a viewer in shared experiences, possibly leading to deficits in learning from video relative to learning from responsive face-to-face encounters. One hundred and seventy-six toddlers (24 and 30 months old) were offered referential social cues disambiguating a novel word’s meaning in one of four conditions: *responsive live* (a speaker was present and engaged with children); *unresponsive video* (a speaker on video looked at the camera and smiled at scripted times); *unresponsive live* (although present, the speaker behaved as she did on the unresponsive video), and *responsive video* (a speaker on closed-circuit video engaged with children, as in video chat). Children of both ages reliably learned the word in the responsive live condition, and older children (30 months) learned in the unresponsive live condition. Neither group learned in the responsive or unresponsive video conditions. The results show that the addition of communicative social cues to the video presentation via video chat was not sufficient to support learning in this case. Rather, toddlers’ transfer and generalization of words presented on video chat may depend on other contextual factors, such as co-viewers who scaffold their learning. Live, responsive video as implemented in this and prior studies is compared, with implications for the use of video chat via the Internet with young children.

## Introduction

In 1968, the groundbreaking program Sesame Street was developed in the belief that television could be used to teach preschool children from lower income families academic skills they often lacked at school entry (Bereiter and Engelman, 1966; Cooney, 2001; Palmer and Fisch, 2001). This proposal was initially met with skepticism (Davis, 2008), but decades of research clearly show that Sesame Street viewers were better prepared for school than non-viewers were (Wright et al., 2001; Zill, 2001) and that early viewing was associated with better educational outcomes as late as adolescence (e.g., Anderson et al., 2001). One specific benefit was that preschoolers’ vocabulary increased after exposure to Sesame Street (Rice et al., 1990).

A more recent development in children’s media has been the introduction of educational videos designed for babies. Many parents evidently believe that, like their older siblings, infants and toddlers who watch videos will learn from them (Guernsey, 2007). In 2016, parents indicated that education was one of their prime motives for allowing their young child (as young as 6 months) to use screen media (Nabi and Krcmar, 2016).

Underlying this development of video to teach infants is the assumption that viewing a video image is akin to experiencing the pictured event directly (Ittelson, 1996). An iconic symbolic medium, video retains much of the information available in unmediated experience (e.g., the color, shape, and motion of objects; the temporal sequence of events – Strouse and Troseth, 2008). With an accompanying soundtrack, video can realistically show people’s behavior, including facial and vocal expressions, gestures, and speech. Live video can show such events in real time. However, a video is still a representation consisting of flat images on a screen. Protracted, age-related development in very young children’s use and understanding of other kinds of symbols (such as pictures, maps, and scale models – e.g., DeLoache, 1987, 1991; DeLoache and Burns, 1994; Liben and Yekel, 1996) foreshadows a need for similar development in using video.

Infants and toddlers are less likely to use information from a TV screen than from a real event to solve problems (Troseth and DeLoache, 1998; Schmitt and Anderson, 2002; Deocampo and Hudson, 2005) or learn new skills (McCall et al., 1977; Barr and Hayne, 1999; Hayne et al., 2003; Strouse and Troseth, 2008). This pattern of results was termed the video deficit by Anderson and Pempek (2005). It is also evident for language learning: toddlers who hear a new label uttered by a speaker on a recorded video are less likely to learn that word than those who hear the same word from an in-person speaker (Krcmar et al., 2007; Krcmar, 2010; Roseberry et al., 2014). Although language learning from video occurs in some situations (e.g., Schafer and Plunkett, 1998; Scofield et al., 2007; Linebarger and Vaala, 2010; Vandewater, 2011), when learning from video is directly compared to learning from face-to-face interactions toddlers usually learn better from direct experience (i.e., an actual event or person who is present).

One important contributing factor may be very young children’s reliance on social cues to support their language learning (Baldwin, 2000; Baldwin and Moses, 2001; Gergely et al., 2007; Richert et al., 2011). To explain what is missing from video, Kuhl et al. (2003) proposed that interpersonal social cues offered in a face-to-face setting “attract infants’ attention and motivate learning” and that the presence of a person allows the sharing of “information that is referential in nature” (p. 9100). A speaker’s communicative intentions may be less clear when offered on video, and the units of language harder to extract (Kuhl, 2007).

A speaker on television (such as Dora the Explorer or Mr. Rogers) can offer referential cues that indicate the focus of his or her attention – such as pointing or gaze direction toward an on-screen entity. Visible on-screen referential cues such as gestures (Rader and Zukow-Goldring, 2010), face direction (Houston-Price et al., 2006), and pointing and nodding (Briganti and Cohen, 2011) are used by infants to support word learning in studies where labels are taught and tested on screen. In these studies, infants were only asked to pair the on-screen label with the on-screen object by looking to the correct part of the screen when the label was spoken. Thus, referential cues provided on screen appear to support infants in *associating* the correct image with its label. However, infants may not be using the referential cues as anything more than an attentional spotlight that does not require them to make an inference about speakers’ intentions. If this is the case, then screen-based referential cues may not provide all of the support that young children need to learn the word robustly enough to generalize, or apply that word to other contexts. In other research that included measures of this type, a video deficit occurred when toddlers were asked to generalize verbs to a new actor (Roseberry et al., 2014), or transfer object labels to real-world objects (Krcmar et al., 2007; Krcmar, 2010).

Generalization and transfer require a more robust understanding of a word’s meaning than simple association between an on-screen object and label (Werker et al., 1998; Horst and Samuelson, 2008; Axelsson and Horst, 2013; Bion et al., 2013; Zosh et al., 2013). Interpersonal cues, or communicative cues shared between the speaker and child, may be important for supporting these processes. From the middle of the first year, infants notice the lack of interpersonal contingency of a person on TV and are more responsive to a person who is present (Bigelow et al., 1996; Hains and Muir, 1996). In several studies, after exposure to a non-responsive (videotaped) person, infants reacted less to a person trying to interact with them via live video, compared to when they saw the live video first (Hains and Muir, 1996; Bigelow and Birch, 2000). This result suggests that the infants no longer expected a person on screen to be engaged with them. People on pre-recorded television can provide at best a “non-contingent, quasi-social” situation for young viewers (Hollenbeck and Slaby, 1979, p. 45) by offering a subset of social cues indicating engagement, such as apparent eye contact (looking directly at the camera), smiling, and the use of infant directed speech. However, they do not respond personally to the viewing child or use his or her name, and they cannot share focus with the child on objects or events in the child’s environment. These communicative cues that typically are shared between a teacher and child may be important for establishing the relevance or applicability of what is being learned and may contribute to the robustness of children’s learning.

According to Wilson and Sperber (2004), an input is relevant to an individual when it connects with their background information to yield conclusions that matter to that person. For instance, preschool children learned the name of a novel toy when told it was “bought downtown” and was for local children, but failed to learn the word when they were told the toy was bought in a faraway country and was special to children there (Henderson et al., 2013). Citing relevance theory, the authors reason that the benefit of knowing the name of a foreign toy was not worth the effort required for young children to learn and store the information. In the same way, learning from a person on video robustly enough to support later generalization of the information beyond the screen might rest on whether children judged the information to be relevant to them.

Would this judgment depend on the person being on a screen *per se*, or would additional interpersonal social cues help children to learn from video? In two independent studies using an object retrieval task, 24-month-olds’ learning assumed the typical “video deficit” pattern: children followed the verbal directions of a person who was present approximately three times as often as when the same person spoke from a TV screen (Troseth et al., 2006; Schmidt et al., 2007). However, in a follow-up study, the person on video offered a full range of interactive social cues though the use of a live feed: she conversed with the parent while the child listened, played “Simon Says” with the child, and responded contingently to whatever the child and parent said and did. The on-screen person also offered information that was “referential in nature” – referring to the child by name, conversing with the parent about the child’s recent birthday or the family pet, and directing the child to a particular object in the real-world environment outside the screen (a sticker in a box under the child’s chair). After interacting with the person on video for 5 min, 24-month-olds readily used the verbal information she provided to solve the problem at a level similar to when they received the information directly from a person in the room (Troseth et al., 2006; also see Nielsen et al., 2008). Thus, there was no deficit relative to *in vivo* learning for this age group when a responsive speaker on a closed-circuit video feed (similar to video chat) provided the information.

Due in part to studies such as these, the influential guidelines published by the American Academy of Pediatrics (Chassiakos et al., 2016) make an exception for video chat in their recommendations that children under age two not be exposed to screen media. Based on this well-publicized recommendation, parents may assume that infants and toddlers will benefit from video chat. However, it is important to know whether the intact social cues possible in live-feed video, by themselves, are sufficient for a speaker to signal communicative intent to a young viewer. With a live video feed, the person on screen provides social cues that are typically available only in a face-to-face interaction: he or she can truly be responsive to the viewing child, refer to the child by name, and engage contingently in shared experiences. Video chat allows children and an on-screen adult to coordinate their attention such that they can share focus on an object or event. It is possible that toddlers will respond to, and learn from, video chat without exhibiting the typical “deficit” in learning (Anderson and Pempek, 2005).

The study reported here adds to the current literature by investigating whether the social cues provided in a labeling session alone (without a specially designed introduction to video chat via live feed) are enough to support children’s word learning. In the challenging word-learning task used here, children needed to use the speaker’s referential cue (gaze into an opaque container while labeling) to learn the word, while avoiding associating the word with a visible object. The presence of a visible distracter required children to use the speaker’s gaze into the opaque container to infer the location of the labeled object, while not connecting the label with the visible object (see Baldwin, 1991, 1993). Learning the novel label from video thus relied on children recognizing the on-screen speaker’s intent to refer to the unseen object; children could not learn the word by association (as when a voiceover of a word repeatedly co-occurs with the isolated image of an object on a video screen – Golinkoff et al., 1987; Scofield et al., 2007).

We used a factorial design to systematically address whether the presence or absence of responsive social cues was necessary or sufficient for toddlers in two age groups to learn the novel word. In one manipulation, the speaker appeared on video or in person. In the other manipulation, the speaker was responsive or unresponsive to the child. This resulted in two conditions consistent with children’s experience interacting with people and watching television – a live, responsive speaker, and an unresponsive speaker on pre-recorded video. It also resulted in two conditions in which these social cues were reversed, i.e., the speaker on TV behaved responsively (as in video chat) and the speaker who was present was unresponsive. In this way, we examined whether for toddlers, seeing a speaker “face to face” made up for missing social cues in recognizing referential intent, and probed whether the presence of basic cues indicating social connection (e.g., use of the child’s name; contingency between the child’s attention and the speaker’s talk) was sufficient to elicit learning from video.

If children learn words from the responsive live and video speakers and not from the unresponsive speakers, it would suggest that toddlers need specific evidence of engagement and relevance to recognize a learning situation. It would support the idea that a lack of social communicative cues partially underlies the video deficit and is the mechanism by which live-feed video has supported learning in prior studies. However, if children learn from the live speakers and not the video speakers, regardless of responsiveness, it would show that communicative cues alone, offered by the person on screen, may be insufficient to support word learning from video and suggest that prior experience or training may be vital to ensure young children’s learning even from live video chat. Importantly, the data reported here were collected before the wide adoption of video chat through the internet. Therefore, participating children did not have such prior experience with video chat and were experiencing a novel situation.

## Materials and Methods

### Participants

Participants were 176 children from a city in the southern United States, divided into a younger group (23 to 26 months; *M* = 24.5 months; 47 male and 41 female) and an older group (29 to 32 months; *M* = 30.5 months; 42 male and 46 female). Recruited from state birth records, participants were mostly Caucasian (95.2%; Black, 3.6%; Asian, 0.6%; no response, 4.6%) and non-Hispanic (95.1%; Hispanic, 4.9%; no response, 18.7%) and came primarily from middle- to upper-middle-class homes. Data from 25 additional children were excluded for non-compliance (12), parental or sibling interference (2), naming a test object during the session (2), speaking a language other than English (1), extended distraction (bathroom break) in the middle of the session (1), and experimenter error (7).

### Materials

**FIGURE 1:**
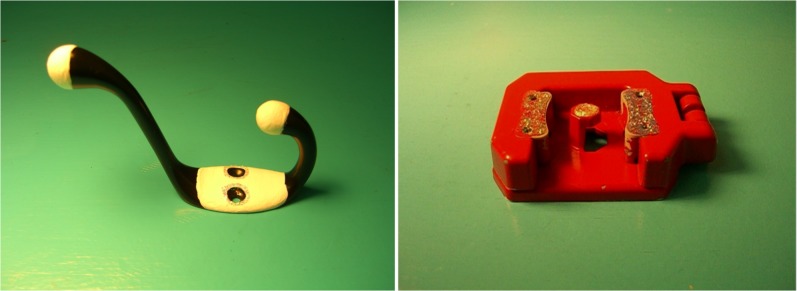
Novel objects. Credit: Photo of stimuli previously included in Strouse et al. (2018).

Children saw two pairs of familiar objects (frog-turtle and boat-truck) and one pair of novel objects (a mop holder and a ceramic hook – see Figure 1). A second set of novel objects, identical to the first in every respect except color, was used to assess children’s generalization of novel labels. Parents were asked whether their child knew the name of the novel objects; for two children, a different object (a handle from a sippy cup) was used. In pilot testing, children of the same age as the participants did not show a preference for any of the objects when they were offered pairs of them.

An opaque plastic bucket and a transparent bin served as containers for the objects during labeling. During testing, children responded by placing chosen objects in a colorful cardboard chute and a transparent plastic tube, which were used to encourage children to respond to test questions. Children sat at a child-sized table, across from a researcher or a 21^′′^ (53 cm) monitor. In the *non-responsive video* condition, a DVD player was used to play a pre-taped video (described below) on the monitor. The sessions were videotaped for later coding.

### Procedure and Design

Children met three researchers during the experiment: the *labeler* labeled the objects during a live or video presentation, the *assistant* kept children on task, and the *tester* administered the comprehension tests. The tester was naïve to which object served as the target because he/she was out of the room when novel labels were offered. For consistency, the labeler was always present during the warm up; in the video conditions, she wore the same shirt as in the video and exited the room while the video played.

Children in both age groups participated in one of the four conditions (responsive or unresponsive live; responsive or unresponsive video). The experiment had four phases: *warm-up, practice, labeling*, and *comprehension*. Across conditions, the procedure was the same except during the labeling phase.

During the 5-min *warm-up* with the tester, children were encouraged to play with the familiar and novel objects, the bucket and transparent bin, and the chute and tube. The tester labeled the familiar objects but offered no labels for the novel ones. Then children were seated across the table from the tester. Parents usually sat by their children, facing away from the tester and working on paperwork.

The function of the *practice* phase was to teach children the researcher’s expectations for the testing procedure: specifically, that they choose the single object requested. The tester extended his/her hands (each holding a familiar object) and asked children to put one item into the chute by using its name (e.g., “Show me the frog. Can you put it in the chute?”), then referred to the other object using a general pronoun (e.g., “What about this one? Can you put it in the chute?”). He/she repeated the sequence using the tube as the apparatus. If children reached for both familiar objects when asked for the target, the tester retracted his/her hands and said, “Just pick one,” before repeating the request. If children chose the wrong object, the tester corrected them and asked them to choose the other item. The entire sequence was repeated for the second pair of familiar objects. The tester always asked about the frog first, but the left–right positions of the targets were counterbalanced.

At the conclusion of the practice phase, the tester left the room. In the live conditions, the labeler took her place at the table across from the child and proceeded with the labeling phase. In the video conditions, the video monitor was placed across the table from the child and the labeler left the room, telling the child, “You’re going to see me on TV!” Then she either proceeded with the labeling over video from an adjoining room (responsive video) or the pre-taped DVD of the labeler was played (unresponsive video).

During the *labeling* phase, the labeler gave children a word for one of a pair of novel objects following the script below. In the two contingent conditions (responsive live and responsive video), the labeler smiled and made eye contact with the child, used the child’s name, and reacted contingently to the child (e.g., by pausing if the child became distracted) while providing the scripted information. In the two non-contingent conditions (non-responsive live and non-responsive video), the labeler smiled and looked toward the child at scripted times, but her actions were not contingent on the child’s behavior nor were they personalized: she did not use their name and she continued smiling and talking whether the child paid attention or was distracted. The non-responsive video labeling demonstration was pre-recorded; the non-responsive live demonstration adhered to the script of the pre-recorded video, and in both cases, the assistant (rather than the non-responsive labeler) used the child’s name and redirected his or her attention as necessary.

To begin the labeling phase, the labeler drew the child’s attention to the target and distracter by holding them both up, then looking at each in turn, saying, “Look at this one!” She then placed the target into the opaque container and the distracter into the transparent container while saying, “Look at what I’m doing! This one goes here, and this one goes here.” Next she looked in one of the containers, offering a novel label about the target (“Here’s a modi! I see a modi! Wow, It’s a modi! I like the modi!”) or matched, enthusiastic utterances about the distracter (“Here’s something! I see it! Wow, here it is! I like it!”). Then she looked in the other container and offered the other utterance. The objects then were removed from and placed briefly in front of their containers. The labeler (on video or in person) removed the toys from the child’s view, and the assistant then offered the child the novel objects to explore. Which object (hook or holder) served as the target, and whether the target or distracter was talked about first, were counterbalanced across children.

Following the labeling phase, the tester returned and began the *comprehension* phase, conducted as described above for familiar objects. The tester asked children to choose the named novel object (the “modi”) four times, twice with the objects presented during the labeling phase (putting them both in the chute and then in the tube) and twice with the set of generalization objects (e.g., “Show me the modi. Pick the modi. Put it down the chute. Can you put the other one down the chute?”). Children were neither corrected nor praised for their responses. Left–right target position and apparatus (chute/tube) used first were counterbalanced.

Parents completed the MacArthur CDI Level II Short Form and a brief questionnaire that included demographic items and information about their child’s media exposure.

### Coding

The labeler or assistant coded children’s comprehension responses on-line. Based on the object children touched first, they received 1 point for selecting the target and 0 for selecting the distracter. These scores were summed across the four trials for a total score between 0 and 4. A second coder, naïve to which object was the target, coded all children’s responses from videotapes. Reliability was high (*κ* = 0.87, *p* < 0.001). Disagreements were resolved by discussion.

To examine whether children were equally attentive to the labeling events across conditions, a master coder watched the videotaped sessions and recorded the number of seconds children spent watching the demonstration (i.e., looking at the monitor screen, or at the real person and objects, depending on condition). We then calculated the proportion of the demonstration each child attended to the labeling event. Due to equipment failure or the child moving off-camera, the session of one child from each age group could not be coded. A second coder recorded looking time for 80% of the sample. The intraclass correlation was *r*(126) = 0.95, *p* < 0.001.

## Results

### Comprehension Test

Preliminary analyses revealed no associations between children’s learning and their vocabulary level or regular weekly television exposure, so these factors were not included in further analyses. There were no differences in children’s responding to test and generalization items, so the scores were totaled across all four word learning trials in the analyses below (similar to other studies using this task – Strouse and Troseth, 2014; Strouse et al., 2018). Children’s mean level of responding to the comprehension questions across age groups and conditions is shown in Table 1. An additional summary table of children’s total scores is provided in the Supplementary Material. Concerns about violations of the normality assumption on several tests were addressed through simple bootstrapping with 10,000 samples (Erceg-Hurn and Mirosevich, 2008). Bootstrapped confidence intervals represent bias-corrected accelerated (BCa) confidence intervals around mean differences. ANOVA results also include bootstrapped *p*-values. Bootstrapped values are indicated with b subscripts.

**Table 1:** Means and standard deviations for Experiment 1 outcome variables.

	Word learning (out of 4)	Percent attention to demonstration
**Responsive live**		
24-month-olds	2.64* (1.40)	87.5% (16.0)
30-month-olds	3.09** (1.41)	95.3% (5.4)
**Unresponsive live**		
24-month-olds	1.95 (1.40)	82.2% (18.6)
30-month-olds	2.95** (1.50)	91.8% (15.9)
**Responsive video**		
24-month-olds	2.23 (1.45)	95.8% (5.0)
30-month-olds	2.55 (1.57)	97.6% (4.0)
**Unresponsive video**		
24-month-olds	2.18 (1.50)	95.9% (5.4)
30-month-olds	2.00 (1.75)	90.2% (14.0)

*Asterisks indicate learning scores significantly different from chance (2 out of 4). *^∗^p* < 0.05. ^∗∗^*p* < 0.01*.

Tests against chance (chance = 2) revealed that children demonstrated reliable word learning from a responsive person who was physically present both at 30 months, *t*(21) = 3.62, *p* = 0.002, 95% CI_b_ = [0.50, 1.64] and at 24 months, *t*(21) = 2.13, *p* = 0.045, 95% CI_b_ = [0.09, 1.14]. When the physically present person was non-responsive, only 30-month-old children displayed reliable word learning, *t*(21) = 2.99, *p* = 0.007, 95% CI_b_ = [0.36, 1.50] (24 months *t*(21) = -0.15, *p* = 0.880, 95% CI_b_ = [-0.64, 0.55]). No reliable learning was observed in either age group when the labeling occurred on video, regardless of whether the labeler was responsive or unresponsive.

We next computed factorial ANOVAs for each age group to identify condition differences in learning, with responsiveness and presentation format (live vs. video) as independent variables. We also included the interaction between responsiveness and format in our model. Despite pilot testing the target objects, we found that children more often chose the correct test object when it was the hook rather than the mop holder. Therefore, we also entered target object (hook vs. mop holder) as a factor in our analyses.

For 30-month olds, there was a main effect of format, such that children learned more from live presentations than video presentations, *F*(1,83) = 5.67, *p* = 0.020, partial eta squared = 0.064, *p*_b_ = 0.027, 95% CI_b_ = [0.09, 1.42]. There was also an effect of which object was the target, *F*(1,83) = 6.15, *p* = 0.015, partial eta squared = 0.069, *p*_b_ = 0.014, 95% CI_b_ = [0.16, 1.44]. There was no significant main effect of responsiveness and no interaction between format and responsiveness. For 24-month olds, no main effects or two-way interactions emerged.

### Attention to Labeling

To investigate differences in the proportion of time that children attended to the labeling event across condition, we carried out a 2 (format: live versus video) × 2 (responsive vs. non-responsive) ANOVA for each age group.

For 30-month olds, there was a main effect of responsiveness, *F*(1,83) = 5.29, *p* = 0.024, partial eta squared = 0.060, *p*_b_ = 0.059, 95% CI_b_ = [0.01, 0.10], such that children were more attentive in the responsive than the non-responsive conditions, although bootstrapped values disagreed on the significance of this effect. There was no effect of format and no interaction. For 24-month-olds, there was a significant main effect of format, *F*(1,83) = 15.81, *p* < 0.001, partial eta squared = 0.160, *p*_b_ = 0.002, 95% CI_b_ = [0.06, 0.17], such that children were more attentive in the live than video conditions. There was no effect of responsiveness, and no interaction. Mean looking times are displayed in Table 1. Attentiveness was relatively high in all cases and the proportion of time children spent attending to the labeling event did not predict how often they selected the correct referent on test trials for either age group, younger: Spearman’s *rho*(85) = 0.08, *p* = 0.487, older: *rho*(85) = 0.16, *p* = 0.148.

## Discussion

The results confirm the presence of a video deficit in word learning for 24- and 30-month-old children. Children of both age groups learned, transferred, and generalized the new label when they heard it given by a responsive, live speaker who was present. When face-to-face with a speaker, children of this age reliably used the speaker’s referential cue (gaze) to distinguish the referent of a novel label, even though that referent was hidden inside an opaque container and a visible distracter was present. In contrast, neither group demonstrated above-chance learning in either video condition. This was the case even when the person on video provided interpersonal communicative cues such as using the child’s name and waiting for children to be attentive before providing information.

When the speaker was present but did not offer interpersonal cues, we found an age-related difference in children’s learning. Thirty-month-olds learned the novel word from a non-responsive speaker who did not address them by name or pause if they became distracted. In contrast, 24-month-olds were at chance in their choices of novel objects as referents for the novel word in the unresponsive live condition, as they were in both video conditions. At 24 months, the word-learning task of using a speaker’s referential cue to identify a hidden referent was difficult if any deviation from normal, face-to-face social interaction occurred, both when these social cues were removed from a face-to-face encounter and when these cues were offered but the speaker was on video. When the cues were removed from face-to-face events, the older toddlers’ learning remained above chance. One possibility is that these older children were as willing to learn from a situation in which an adult was or was not clearly interacting with them due to greater experience in group settings (e.g., in daycare) in which conversation often is overheard rather than directed personally at them. Children of this age robustly learn from overhearing conversational partners talk to and direct referential cues toward each other (e.g., Akhtar et al., 2001; Akhtar, 2005) including when such conversations appear on video (O’Doherty et al., 2011).

Even though the responsiveness manipulation did not result in significant differences in learning for the older toddlers, they paid somewhat more attention in the responsive than the non-responsive conditions. Thirty-month-olds may be starting to identify interpersonal responsiveness as a cue that information is important to attend to. In another study using the same word learning task, children were more responsive to requests for action (like touching their shoulder) from a responsive speaker on video chat than from a person who made the same requests on pre-recorded video, but they did not subsequently learn more from the responsive speaker (Strouse et al., 2018). Although visual engagement with video is not always associated with learning in short lab studies (e.g., Kirkorian et al., 2016), if responsive video chat engages children, it seems likely to support learning from live video over time.

In contrast to the age-related differences in learning from a non-responsive person who was present, neither group of toddlers learned the novel word from video in our challenging labeling task. Children in the video conditions needed to transfer the label they heard while being exposed to two-dimensional depictions on video to the real three-dimensional objects they were tested with (identical objects and generalization objects). Children in the two live conditions saw the same three-dimensional objects during the labeling session and at test, followed by same-dimension generalization objects. In several studies, infants and toddlers more often struggled to put a puzzle together after a demonstration when they needed to transfer from 2D to 3D, or vice versa, than when the format of the objects was the same at both demonstration and test (Zack et al., 2009, 2013; Moser et al., 2015). Children in our live conditions may have been presented with an easier task because success did not require cross-dimension transfer – the definition of learning from a screen and applying that information in the real world. In two recent studies that have reported word learning from video chat, children held real objects as identical ones were being labeled on screen (Myers et al., 2017, 2018), and the authors acknowledge that their test of word learning did not involve cross-dimensional transfer. A study of verb learning from video chat also did not involve video-to-real-world transfer, as both the labeling events and the test events appeared on video (Roseberry et al., 2014).

A question that remains is what circumstances are necessary and sufficient for children’s learning from video chat. The influential guidelines published by the American Academy of Pediatrics (Chassiakos et al., 2016) make an exception for video chat in their recommendations that children under age two not be exposed to screen media. Therefore, parents may assume that infants and toddlers will benefit from video chat – that they automatically will understand and learn from it. However, very young children may need help to interpret the social cues offered by a responsive person on screen. In two recent word-learning studies, toddlers were more likely to learn words from video chat when they watched the labeling demonstration alongside a co-viewer who had modeled responsiveness to the on-screen actress (Myers et al., 2018; Strouse et al., 2018). With time and experience, young children might figure out the relation between live video and reality by themselves, although children’s prior exposure to video chat has not been related to outcomes in any of the studies published thus far. It therefore is important to know how scaffolding by an adult co-viewer might speed up this process and ensure children’s understanding, particularly if parents now believe that toddlers will easily learn from video chat.

Research to date indicates that both adults on video chat and adult co-viewers are influential for children’s interpretation of screen-based events as social interactions. McClure et al. (2018) use the term within-screen joint visual attention to refer to instances in which a person engaging in video chat calls attention to something on their own side of the screen (e.g., the adult points out something in their own environment) and across-screen joint visual attention to refer to instances in which someone directs attention to something in another person’s environment (e.g., the on-screen adult points out something in the viewing child’s environment). McClure et al. (2018) observed infants 6 to 24 months interacting through video chat with a grandparent on screen and a parent present in the room. They found that the amount of both within-screen and across-screen joint attention that mothers (co-viewers with the child) initiated were positively associated with the amount of joint attention the child initiated, and the type of joint attention mothers tended to initiate matched the type their children tended to initiate. They also found that older infants more frequently initiated joint attention, especially across-screen joint attention, than younger children. Their findings are correlational, but one explanation is that children learn to initiate across- and within-screen joint attention by engaging with co-viewers who model the behavior, as well as on-screen adults who respond to the child’s bids for screen-mediated joint engagement. It is possible that children learn to engage with others via video chat through their scaffolded experiences with the medium.

The idea that experience, training, or co-viewer support is needed to scaffold children’s learning from video chat is consistent with prior studies, in which children may have learned from live video feeds because of the thoughtfully designed and well-scaffolded training sessions that they received prior to watching demonstrations of the to-be-learned information. For example, the on-screen adult in these studies referred to personal details about the child such as their name or siblings (Troseth et al., 2006; Nielsen et al., 2008; Roseberry et al., 2014; Myers et al., 2017), directed attention to a specific object in the child’s environment and asked the child to retrieve it (Troseth et al., 2006; Nielsen et al., 2008), asked the child to participate in interactive games such as pointing to her own nose and asking the child to do the same (Troseth et al., 2006; Roseberry et al., 2014; Myers et al., 2017), and interacted with the co-viewing parent while the child observed (Troseth et al., 2006; Nielsen et al., 2008). These interactive training sessions were intended to “establish the experimenter as a trusted source of information” and “demonstrate the interactive nature of video chat” (Roseberry et al., 2014, p. 961). In the current research, we did not include training prior to the live video labeling demonstration; only simple evidence of contingent responsiveness was provided, such as use of the child’s name and waiting for the child’s attention before continuing to talk. Children did not reliably learn and generalize the new word in these circumstances. Thus, prior experience with video chat, particularly combined with active co-viewer support, may be important for toddlers to realize that information presented on video chat is relevant to life outside the screen, and should be generalized and transferred.

Other methodological differences between the current research and studies that reported word learning from video chat are informative. Besides the lack of cross-dimensional transfer mentioned above, the studies involved more repetitions of each novel label (12 repetitions – Roseberry et al., 2014; 18 repetitions – Myers et al., 2017). Children in these studies observed actual, full-screen Skype calls that started with the characteristic Skype ringtone, rather than the closed-circuit video used in the current study. In the research by Myers et al. (2017), children experienced an initial Skype call between rooms in the lab, and then five additional calls between the lab and home during the next 2 weeks, giving them substantial prior experience video chatting with a responsive speaker who labeled objects on screen that were the same as those the children held in their own hands.

Gaze alignment of the on-screen person and the viewer also differs across the studies, as a result of the different camera and display setups used. Unlike the mis-aligned gaze typical of real-world video chat and closed-circuit video (such as that used in our study), Myers et al. (2017) aligned the on-screen person’s gaze direction toward the viewing child through the use of a camera attached to the center of the speaker’s video screen (so that the camera was close to the child’s eye location on that screen), and children successfully learned words. In Myers et al.’s (2018) next study, gaze alignment was systematically varied and did not significantly impact word learning – children learned in both conditions as long as they had a responsive co-viewer. In Roseberry et al.’s (2014) verb-learning study, the more that children looked at the on-screen partner’s eyes during the interactive, responsive training session, the more novel verbs they learned – even though the person’s gaze at them appeared mis-aligned. Therefore, differences in the alignment of gaze direction to the viewer do not seem likely to explain differences in word learning across studies. It should be noted that in all of these studies (including the current one), the on-screen person’s gaze *toward the referent object or action* had accurate alignment.

One additional explanation for children’s pattern of behavior in our study is that success at our task may involve children’s expectations about whether people on video typically provide relevant information, so that a speaker’s referential cue of gaze at the named object would have meaning for the viewer (as is the case with people who are actually present – Baldwin and Moses, 2001). As mentioned earlier, the current research was conducted before video chat via the internet was widely available, so the children did not enter the study with prior experience. On the other hand, almost all of the children (92%) had regular weekly exposure to pre-recorded video on television and those few who did not were distributed across condition. From being exposed to television, children learn that a person talking on TV does not share focus with them on elements of their environment or provide information related to their ongoing experience (Troseth, 2003; Troseth et al., 2006). In contrast, children’s experience in face-to-face interaction may lead them to expect conversational partners to share information relevant to the listener (following Grice’s principles of cooperation and relation/relevance – Wilson and Sperber, 2004) barring evidence of untrustworthiness (e.g., a partner who mislabels familiar objects – Koenig and Echols, 2003). As more experienced social communicators, older toddlers may have this expectation even more firmly established than younger toddlers do.

In accumulating knowledge of the world (including the names of things), toddlers are thought to develop experience-based initial conceptions that affect their information processing, such as gender schemas (e.g., Bauer, 1993; Ruble and Martin, 1998) and biases regarding word learning (Hollich et al., 2000; Imai and Haryu, 2004; Saylor and Sabbagh, 2004). For instance, according to gender schema theory, children pay less attention to information they determine does not relates to their gender because they view such information as not relevant to themselves. Similarly, the “video deficit” – relatively inefficient learning from video versus real situations – may result in part from toddlers’ expectation that events on the screen do not relate to their lives in the real world. Thus, support to help very young children learn from video chat may involve adult co-viewers pointing out, illustrating, and modeling the connection between events on screen and current reality. For example, in a study using the same word learning paradigm, more children learned the word when parents simply pointed out that the objects on TV were the same as those in the room (Strouse and Troseth, 2014).

## Conclusion

We conclude that a lack of communicative cues given during labeling are not the sole reason for video deficits in word learning, and the presence of such on-screen cues is not the full explanation for children’s success in prior studies using live video feeds. Video chat appears to engage children, but more research will clarify how children come to understand and learn from video chat experiences. Learning from video (even live, responsive video) is challenging for young children, and co-viewer support may provide a necessary scaffold.

## Data Availability Statement

The raw data supporting the conclusions of this manuscript will be made available by the authors, without undue reservation, to any qualified researcher.

## Ethics Statement

This study was carried out in accordance with the recommendations of Vanderbilt University & Institutional Review Board with written informed consent from all subjects. All parents of children who participated gave written informed consent, and all participating children gave verbal assent, in accordance with the Declaration of Helsinki. Children were given the option to cease participation at any time if they did not want to continue.

## Author Contributions

GT and MS developed the study materials and procedures. GS and BV collected the data, provided guidance in the development of the project, oversaw the transcription and coding of data, and analyzed and interpreted the data. GT and GS wrote the manuscript. BV and MS provided feedback on and edits to the manuscript.

## Conflict of Interest Statement

The authors declare that the research was conducted in the absence of any commercial or financial relationships that could be construed as a potential conflict of interest.
