# Corrigendum: Let's Chat: On-Screen Social Responsiveness Is Not Sufficient to Support Toddlers' Word Learning From Video

**DOI:** 10.3389/fpsyg.2019.01793

**Published:** 2019-08-08

**Authors:** Georgene L. Troseth, Gabrielle A. Strouse, Brian N. Verdine, Megan M. Saylor

**Affiliations:** ^1^Department of Psychology and Human Development, Vanderbilt University, Nashville, TN, United States; ^2^Division of Counseling and Psychology in Education, University of South Dakota, Vermillion, SD, United States; ^3^School of Education, University of Delaware, Newark, DE, United States

**Keywords:** contingency, relevance, social cues, symbols, video, video chat, word learning

In the original article, there was an error. In the Abstract, the number of participants was mistakenly listed as “One hundred and thirty-two toddlers,” an outdated number from a previous version of this paper, instead of “One hundred and seventy-six” (176).

A correction has been made to the ***Abstract***:

“Joint engagement with a speaker is one cue children may use to establish that an interaction is relevant to them and worthy of attention. People on pre-recorded video cannot engage contingently with a viewer in shared experiences, possibly leading to deficits in learning from video relative to learning from responsive face-to-face encounters. One hundred and seventy-six toddlers (24 and 30 months old) were offered referential social cues disambiguating a novel word's meaning in one of four conditions: responsive live (a speaker was present and engaged with children); unresponsive video (a speaker on video looked at the camera and smiled at scripted times); unresponsive live (although present, the speaker behaved as she did on the unresponsive video), and responsive video (a speaker on closed-circuit video engaged with children, as in video chat). Children of both ages reliably learned the word in the responsive live condition, and older children (30 months) learned in the unresponsive live condition. Neither group learned in the responsive or unresponsive video conditions. The results show that the addition of communicative social cues to the video presentation via video chat was not sufficient to support learning in this case. Rather, toddlers' transfer and generalization of words presented on video chat may depend on other contextual factors, such as co-viewers who scaffold their learning. Live, responsive video as implemented in this and prior studies is compared, with implications for the use of video chat via the Internet with young children.”

The authors apologize for this error and state that this does not change the scientific conclusions of the article in any way. The original article has been updated.

