# Supplementary material for: Let’s Chat: On-Screen Social Responsiveness Is Not Sufficient to Support Toddlers’ Word Learning From Video
Source: Front Psychol. 2018 Nov 13;9:2195. doi: 10.3389/fpsyg.2018.02195 (PMC6243085; doi:10.3389/fpsyg.2018.02195)
Supplement: Supplementary file 1 [file Table_1.docx]

Supplementary Material

*Number of Children Receiving Each Total Score Across the 4 Word Learning Test Trials.*

|  | Total Word Learning Score | | | | |
| --- | --- | --- | --- | --- | --- |
| Group | 0 | 1 | 2 | 3 | 4 |
| 24 months responsive live | 3 | 2 | 2 | 8 | 7 |
| 24 months unresponsive live | 5 | 3 | 5 | 6 | 3 |
| 24 months responsive video | 4 | 2 | 7 | 3 | 6 |
| 24 months unresponsive video | 4 | 4 | 4 | 4 | 6 |
| 30 months responsive live | 2 | 2 | 2 | 2 | 14 |
| 30 months unresponsive live | 3 | 1 | 3 | 2 | 13 |
| 30 months responsive video | 4 | 2 | 3 | 4 | 9 |
| 30 months unresponsive video | 8 | 1 | 3 | 3 | 7 |
